# Glucose-Reduced Graphene Oxide with Excellent Biocompatibility and Photothermal Efficiency as well as Drug Loading

**DOI:** 10.1186/s11671-016-1423-8

**Published:** 2016-04-19

**Authors:** Hongyu Liu, Tan Li, Yuhong Liu, Guiqi Qin, Xiaoping Wang, Tongsheng Chen

**Affiliations:** MOE Key Laboratory of Laser Life Science & College of Biophotonics, South China Normal University, Guangzhou, 510631 China; Department of Pain Management, The First Affiliated Hospital of Jinan University, Guangzhou, 510630 China

**Keywords:** Nitronium oxidation, Functionalized graphene oxide, Glucose reduction, Biocompatibility, Photothermal effect, Drug loading

## Abstract

In the present work, we report a facile and rapid green strategy to fabricate functionalized reduced nano-graphene oxide (nrGO) as a cooperative nanotemplate for both photothermal therapy and drug loading. Graphite oxide was firstly oxidated by nitronium ions (NO_2_^+^) solution at the aid of microwave heating to obtain nano-GO (nGO) with about 50 nm of diameter, and the nGO was then reduced in pure glucose at 135 °C for 30 min to obtain nrGO with about 40 nm of diameter. The nrGO exhibits excellent biocompatibility including stable dispersibility in cell culture medium and rapid cellular uptake as well as non-cytotoxicity up to 100 μg/mL. Absorption of the nrGO at 808 nm wavelength increased more than 10-folds compared with nGO. Moreover, the nrGO has the ability to load about 317 % (*w/w*) of doxorubicin (DOX), and the loaded DOX could be effectively released by acid condition and/or glutathione (GSH) and/or heating. Finally, a greater cancer cell death efficacy was observed in nrGO/DOX-treated cells with GSH and heating stimulation respectively or their combination. Collectively, the nrGO developed here is an outstanding cooperative nano-platform for high-efficiency photothermal therapy and controllable drug delivery.

**Electronic supplementary material:**

The online version of this article (doi:10.1186/s11671-016-1423-8) contains supplementary material, which is available to authorized users.

## Background

Graphene oxide (GO) has been widely used as an alternative and promising photo-absorbing agent for photothermal therapy (PTT) due to its high photothermal responsiveness, low toxicity, and low cost [1–3]. On the basis of ultrahigh surface area and polyaromatic structures, GO is also available for efficiently loading aromatic hydrophobic drugs via hydrophobic interaction and π-π stacking [4–6]. Reduced GO (rGO), owing to the considerably enhanced optical absorbance and the greatly restored conjugated structures, is more efficient in PTT for large tumors or tumors deeply located inside the body at relatively low power of near-infrared (NIR) laser irradiation and also exhibits preferable drug carrying capacity [7–10].

Due to the mild reduction ability, easy accessibility, abundant oxidative groups composition, and nontoxic merit, glucose is considered as an excellent green candidate for the reduction of GO [11–14]. Zhu and co-workers for the first time used glucose as reductant to reduce GO, and the reaction was carried out at 95 °C for 60 min in the presence of reducing sugar and ammonia solution [11]. The ammonia solution was used as catalyst to accelerate the reduction reaction synergistically and benefit the deoxygenation of GO, and it was illustrated that such reducing capability was closely related to the ability of the saccharides to form open-chain structures [15]. Iron (Fe) was also used as catalyst to accelerate the electron transfer between GO and glucose [14]. In addition, Yuan and co-workers reduced GO in glucose and ammonia solution under 95 °C for 2 h to obtain rGO with increased specific surface area and enormous micro- and meso-pore [12], and Shen and co-works reduced GO in this solution with different fractions of glucose and ammonia in an autoclave under 160 °C for 4 h to obtain rGO with 4.59 of C/O ratio [13]. However, these synthetic materials become sediments after centrifugation, thus they are too heavy and unsuitable for loading abundant drugs. Furthermore, both catalyst and bacteria must be removed before cell application.

In this work, we developed a novel green route to reduce nano-GO (nGO) by adopting pure glucose as a reductant to obtain reduced nano-graphene oxide (nrGO). Prior to the reduction, nGO was prepared by a simple microwave-enabled nitronium oxidation on graphite oxide. The resulting nrGO has three marked advantages: (1) excellent biocompatibility: very stable for at least 1 month in both water and cell culture medium (DMEM); (2) effective photothermal effects: ~10.2-fold increment in NIR absorption at 808 nm compared with the unreduced nGO; and (3) high drug loading capability: about 317 % (*w/w*) of loading capacity for doxorubicin (DOX). In addition, loaded DOX can be effectively released by acid condition and/or glutathione (GSH) and/or heating. These characteristics make the nrGO developed here a very excellent cooperative nano-platform for high-efficiency PTT and controllable drug delivery.

## Methods

### Synthesis of nGO

Graphite oxide was prepared by a modified Hummer’s method utilizing expandable graphite flake (XF NANO Co., Ltd. China). For reoxidation, 12 mg of graphite oxide was mixed with nitronium ion solution in a microwave reaction kettle (Xi’an Often Instrument Equipment Co. Ltd.) for 20 s, and then the entire mixture was placed into a microwave reactor chamber (Midea, MM823LA6-NS) to be heated for 3.75 min at the power of 160 W. After the reactant was cooled by ice bath, the reaction was quenched with 100 mL of deionized water, neutralized by NaOH and Na_2_CO_3_, and ultrafiltered repeatedly through a 30-kDa filter (Millipore) to remove the inorganic salt. At last, the graphite oxide of reoxidation was sonicated (Xin Zhi, JY92-2D) at 612 W for 1 h in an ice bath to obtain nGO. nGO-0, as a control, was fabricated by sonication (Xin Zhi, JY92-2D) of graphite oxide flake at 612 W for 1 h in an ice bath as described previously [9].

### Synthesis of nrGO

After 110 mg glucose was added to 5 mL nGO suspension (about 1 mg/mL) and sonicated for 30 min, the mixture was transferred to autoclave and react at 135 °C for 30 min to achieve sterile nrGO. The resulting nrGO was stored at 4 °C for further use. nrGO-0 was prepared by reducing nGO-0 similarly to the synthesis of nrGO.

### Characterization of nrGO

The sheets of nGO-0, nGO, nrGO-0, and nrGO were imaged with atomic force microscopy (AFM, Agilent Technologies 5500, USA) on a mica substrate. UV-Vis spectra were performed using a UV-Vis spectrometer (Lambda 35, Perkin-Elmer, Waltham, MA, USA) with a 1-cm quartz cuvette. Absorption spectra were measured by an auto microplate reader (Infinite M200, Tecan, Austria). Fourier transform infrared (FTIR) spectra were recorded on a FTIR spectrometer (Bruker Tensor 27, Karlsruhe, Germany). To prepare nrGO samples without non-covalently bound glucose for FTIR spectrometer assay, NaCl was added to nrGO solutions to precipitate nrGO irreversibly, and then the precipitation was centrifuged and rinsed ten times. Raman spectra were taken with a Renishaw (New Mills, UK) inVia micro-Raman spectroscopy system equipped with a 514.5-nm Ar^+^ laser. Luminescence Spectrometer (LS 55, PerkinElmer, USA) was used to measure fluorescence emission of DOX with 488 nm excitation. The images of all samples were recorded using a digital camera (Nikon, Tokyo, Japan) with 1280 × 1280 pixels resolution. The measurements of size distribution, Zeta potential, and polydispersity index (Pdi) of GO materials were conducted with the Zetasizer (Malvern Instruments Ltd., UK), in which mean size = size class (nm) × number distribution date (%).

### Photothermal Irradiation

Eight-hundred-eight-nanometer laser with the power density of 3 W/cm^2^ was used for NIR irradiation. Solutions in Eppendorf tubes were continuously irradiated by 808 nm NIR laser for 8 min, and temperature was measured by a thermocouple thermometer (Fluke 51II, USA) every other 1 min. All the experiments were conducted at room temperature. All experiments were performed in triplicate.

### Loading and Release of DOX

To remove hydrogen chloride from doxorubicin hydrochloride salt (DOX · HCl), 1.3 equivalent of triethylamine was added in DOX · HCl (16 mg) to DMSO (200 μL). After being stirred for 12 h, the solution was filtrated to remove the insoluble triethylamine hydrochloride and the resulting DOX solution (80 mg/mL) was stored at 4 °C. The whole procedures were implemented in the dark [16, 17].

Loading of DOX on nrGO was carried out by adding DOX dissolved in DMSO (13.33 mg/ml) to nrGO aqueous suspension (0.1667 mg/ml) with shaking for 12 h. The final volume ratio of DMSO to H_2_O was 0.5:10. The product was repeatedly filtered through 30 kDa filters (Millipore), soaked in distilled water to remove free DOX and DMSO, and centrifuged at 6000 rpm to remove undissolved drug. DOX was loaded on nrGO-0 in the same way. The loading capacity of DOX on nrGO was estimated by the auto microplate reader from the absorbance at 488 nm, deducting the absorbance of nrGO.

The amount of DOX released from nrGO/DOX under different kinds of condition was estimated by measuring the fluorescence at 604 with 488 nm laser excitation. The solution with DOX-loaded nrGO (0.01 mg/mL) was incubated at different conditions as diverse as a range of GSH concentrations (0, 0.01, 0.05, 0.2, 0.5, 1, and 5 mM), acidic condition (pH 5), or 50 °C water bath for predetermined times to accelerate the embedded drugs release. The amount of DOX release was evaluated by measuring the DOX fluorescence via the auto microplate reader.

### Cell Culture

HepG2, Huh7, and A549 cell lines obtained from the Department of Medicine, Jinan University (Guangzhou, China) were cultured in Dulbecco’s modified Eagle’s medium (DMEM, Gibco, Grand Island, USA) supplemented with 10 % fetal calf serum (FCS) in 5 % CO_2_, 95 % air at 37 °C in a humidified incubator.

### Cell Uptake Assay

After A549 cells (1 × 10^5^ cells) cultured in 6-well plates with 1.5 mL DMEM for 24 h were incubated with nrGO/DOX (5 μg/ml of DOX equivalent) in DMEM (10 % fetal calf serum) for 1, 2, 6, and 12 h, respectively, the cells were rinsed by phosphate-buffered saline (PBS) five times, centrifuged, and resuspended in PBS. The uptake of nrGO/DOX by A549 cells was measured by flow cytometry (FCM, FACSCantoII, Becton Drive, NJ, USA), and 10,000 events were recorded for each FCM analysis. In addition, fluorescence microscope (IX73, Olympus, Japan) with 488 nm excitation and 510–550 nm emission was used to image the uptake of nrGO/DOX by A549 cells.

### Cytotoxicity Assay

For cytotoxicity assay, ~5000 cells/well were plated in 96-well plate with 100 μL DMEM and cultured for 24 h. After incubation with various concentrations of nrGO in DMEM (10 % fetal calf serum) for another 24 h, the relative cell viability was assessed by Cell Counting Kit-8 (CCK-8, Dojindo, Japan) assay with the auto microplate reader as described previously [18].

To exclude the absorbance of nrGO at 450 nm, every treatment group contained six wells, among which the former three wells were treated by CCK-8 reagent whereas the later three wells by DMEM alone, and the differences between the former three wells and the later three wells were considered as the absorption value of CCK-8 reagent. All experiments were performed in triplicate.

For photothermal therapy, adherent cells were incubated with various concentrations of nrGO for 3 h and then irradiated by 808 nm laser for 5 min, and incubated for an additional 21 h before CCK-8 assay.

Cytotoxicity of nrGO/DOX was also evaluated by CCK-8 assay. The cells were allowed to adhere to a 96-well plate overnight before adding nrGO/DOX. Cells were treated with nrGO/DOX to take in for 4 h in 100 μL cell culture medium firstly. The cells with GSH stimulation were treated by GSH at the concentration of 1.5 mM for 2 h, and the cells with 45 °C heating stimulation were shaked in a shaking table at 45 °C for 1 h. After 2 h of drug release treatment, the medium was replaced by fresh medium and the cells were incubated for a further 24 h for CCK-8 assay.

### Statistics

Data were presented as mean ± SD from at least three independent experiments. Statistical and graphic analyses were done using the software SPSS 19.0 (SPSS, Chicago) and Origin 8.0 (OriginLab Corporation).

## Results and Discussion

### Synthesis and Characterization of nrGO

**Scheme 1:**
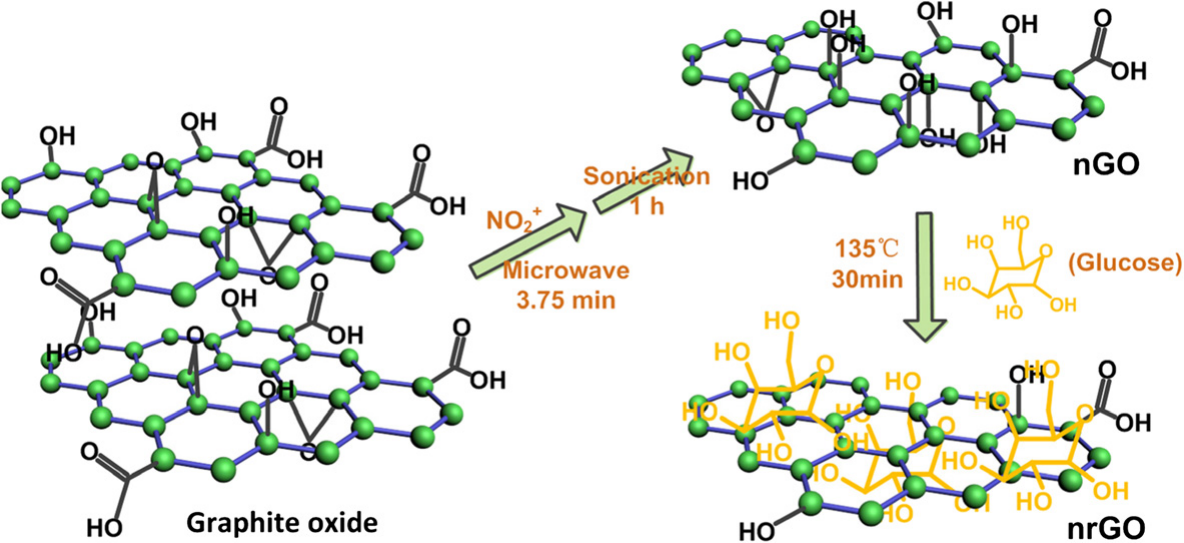
Schematic illustration of the preparation of reduced nano-graphene oxide (nrGO) through nitronium oxidation and glucose reduction

**Fig. 1:**
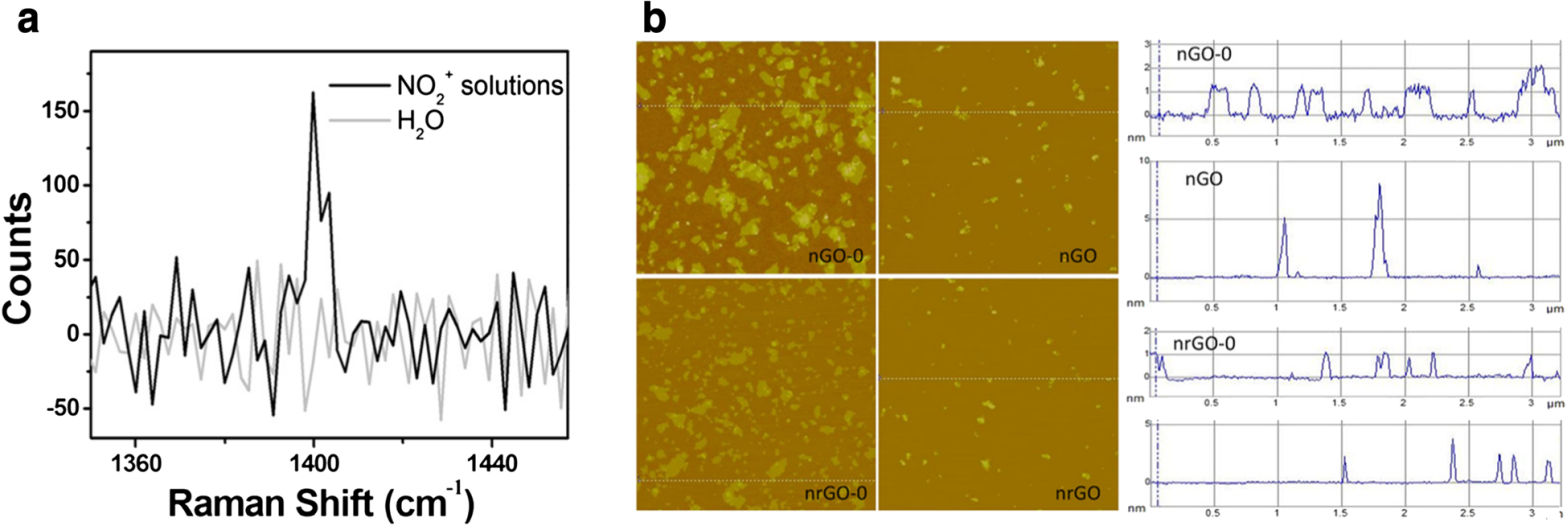
**a** Raman spectra of NO_2_^+^ in water and nitronium oxidation solution. **b** AFM images of nGO-0, nGO, nrGO-0, and nrGO

As shown in Scheme 1, to improve the stability of the final product in physiological solution, we firstly oxidated the aromatic structure of graphite oxide by nitronium ion (NO_2_^+^) at the aid of microwave irradiation for 3.75 min and then sonication for 1 h to achieve nanosized graphene oxide (nGO). Oxidation solution was prepared by mixing 0.7 mL H_2_O, 4.2 mL concentrated H_2_SO_4_, and 0.1 mL concentrated HNO_3_ orderly. Nitronium oxidation was selected to reoxidate graphite oxide in consideration of its simple process, easy elimination of nitro, and much gentle oxidation level on graphite oxide surface over the Hummer method [19, 20] as well as the global oxidation involved the carbon atom within both conjugated structure and oxygen-containing moieties [21–25]. Figure 1a showed the relative NO_2_^+^ concentration, being indicated by the Raman characteristic peak counts at 1394 cm^−1^ from υ(NO) stretching [26], in NO_2_^+^ oxidation solution and water, respectively. Graphite oxide was oxidated by nitronium oxidation solution to achieve nGO with a high yield of 100 %. nGO-0 was fabricated as control by directly sonicating graphite oxide in an ice bath at 612 W for 1 h. The sizes of nGO-0 and nGO were about 150 and 50 nm, respectively (Fig. 1b), indicating that nitronium oxidation made the substance more vulnerable to sonication.

The nGO was next reduced in pure glucose to restore the conjugated structure and enhance the photothermal effect (Scheme 1). Reaction was carried out by heating the nGO and glucose mixture with 1:22 of weight ratio in autoclave at 135 °C for 30 min to obtain reduced nGO (nrGO). The sizes of nrGO-0 and nrGO were about 70 and 40 nm, respectively (Fig. 1b), smaller than that of nGO-0 and nGO, revealing that the chemical bond between graphene sheets may be broken during the reduction process. The sheet thickness of nGO-0, nGO, nrGO-0, and nrGO were approximately 1, 4, 1, and 2 nm, respectively, indicating the bi-layer sheet of nrGO.

**Fig. 2:**
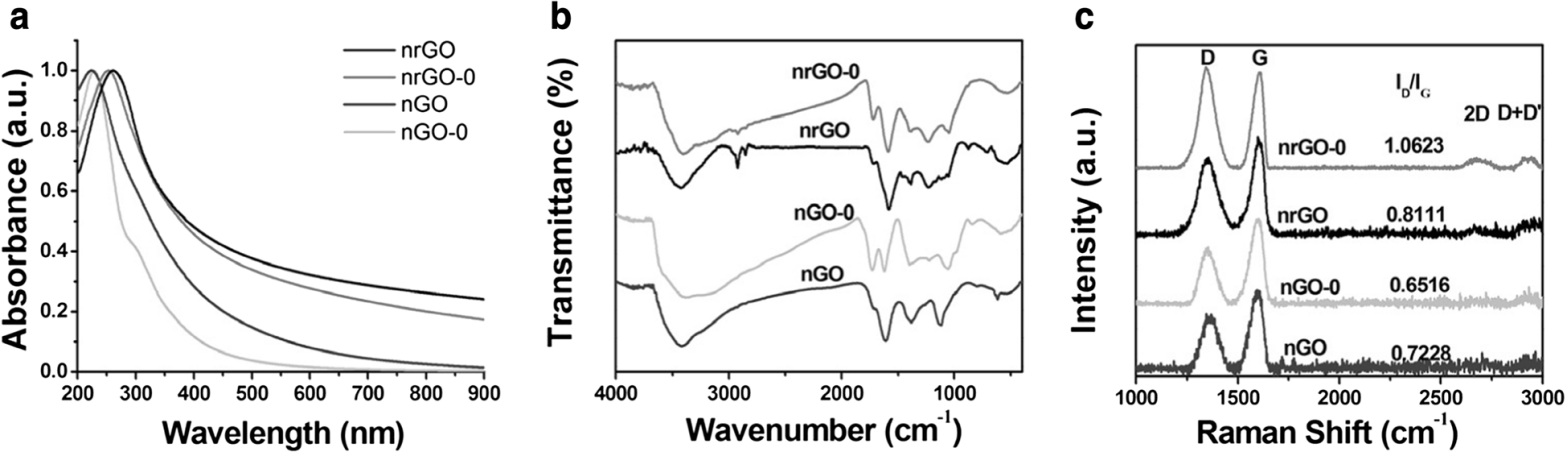
Spectroscopy characterization of nrGO. **a** UV-Vis spectra of nGO and nrGO. **b** FTIR spectra of nGO and nrGO. **c** Raman spectra of nGO and nrGO

The UV-Vis spectra of nGO and nrGO suspensions were exhibited in Fig. 2a. The absorption peaks of nGO had a blue shift from 229 nm (nGO-0) to 224 nm due to the destruction of the conjugated structure upon nitronium ion oxidation. On the other hand, the increase in visible light absorption of nGO indicated an exfoliation of graphite oxide during microwave heating and nitronium oxidation [27–29]. The absorption peaks of nrGO-0 and nrGO shifted to 253 and 261 nm, respectively, and their absorption at 808 nm wavelength increased more than 40.2- and 52.8-folds, respectively, compared with that of nGO-0 (Fig. 2a), demonstrating the restoration of conjugated structure and the better reduction of nrGO than nrGO-0. The nrGO has more than a 10.2-fold increase in absorption at 808 nm wavelength over the unreduced nGO (Fig. 2a). Patel and co-workers have demonstrated that when reacted with graphite, NO_2_^+^ can attack the defect-free graphene planes and etch the existing oxidized sites [25]. It was also revealed that during nitronium oxidation on graphite, multiple hydroxy and/or epoxy groups were formed across the surface of the achieved graphene, and subsequent oxidation resulted in more hydroxy and epoxy groups which were preferentially formed away from the carbon atoms already oxidized due to the electron-donating capability of the resulting hydroxy and epoxy groups [30–32]. We thus speculated that when attacked by NO_2_^+^, the original defects on graphite oxide were consumed and the recovered graphite oxide was covered with a set of elementary oxidation groups, such as hydroxy or epoxy groups, making nGO more sensitive to the reduction conducted by aldehyde groups from glucose.

FTIR spectroscopy is an important tool for the structural characterization of GO-related materials [33]. For nGO and nGO-0, the presence of intense bands at around 3400 cm^−1^(O-H), ~1720 cm^−1^ (C = O), ~1620 cm^−1^ (C = C), ~1220 cm^−1^ (epoxy C-O), and ~1050 cm^−1^ (C-O) (Fig. 2b) indicated the existence of oxygen-containing moieties such as carbonyl, carboxylic, epoxy, and hydroxyl. In the FTIR spectrum of nGO, the reduced absorption at approximately 1720 cm^−1^ due to the C = O stretching and the replacing of the peaks at around 1220 and 1050 cm^−1^ (nGO-0) by a novel type of C-O bonds at around 1110 cm^−1^ (Fig. 2b) also indicated the consumption of original oxygen-containing groups on graphite oxide surface upon nitronium oxidation. Since the unbonded glucose can disperse in NaCl aqueous solution, we added NaCl to nrGO solutions, and then centrifuged and rinsed the precipitation ten times to obtain nrGO without non-covalently bound glucose [34]. The resulting nrGO was subjected to FTIR spectroscopy assay. Compared with nGO, the enhanced peaks at ~1580 cm^−1^ of nrGO (Fig. 2b) suggested a restoration of the carbon basal plane. The greatly reduced peak at about 1230 and 3400 cm^−1^ of nrGO (Fig. 2b) indicated the removal of the epoxy group and hydroxy. On the other hand, the presence of the peaks at ~2920 and ~2850 cm^−1^ corresponding to the symmetric and antisymmetric stretching vibrations of the CH_2_ group and the enhancement of the peaks at ~1720 cm^−1^ attributed to the C = O stretching vibration (Fig. 2b) demonstrated that a part of glucose was covalently connected onto the surface of nrGO.

The Raman spectrum of graphene is characterized by two main features: the G peak arising from the high-frequency E_2g_ phonon at the Brillouin zone center (usually observed at ~1575 cm^−1^) and the D peak arising from the defects-activated breathing modes of six-atom rings (~1350 cm^−1^). The G peak intensity (I_G_) is proportional to the sample area, ∝ L_a_^2^ (L_a_ refers to the crystal size), whereas I_D_ is proportional to the overall length of the edge, which scales as L_a_, thus, the D/G intensity ratio (I_D_/I_G_) varied inversely with L_a_ [35, 36]. As shown in Fig. 2c, the I_D_/I_G_ ratio of nGO is higher than that of nGO-0, indicating the increase in both defects and edges derived from nitronium oxidation. However, the I_D_/I_G_ ratio of nrGO-0 is lower than that of nrGO, indicating that nitronium oxidation of graphite oxide made the resulting nGO particles easier to be reduced and also have fewer defects. However, the reductions of nGO were incomplete as the D + D’ peaks at about 2920 cm^−1^ existed throughout the process, which may be beneficial for the suspension of nrGO.

### Biocompatibility and Photothermal Effect

**Fig. 3:**
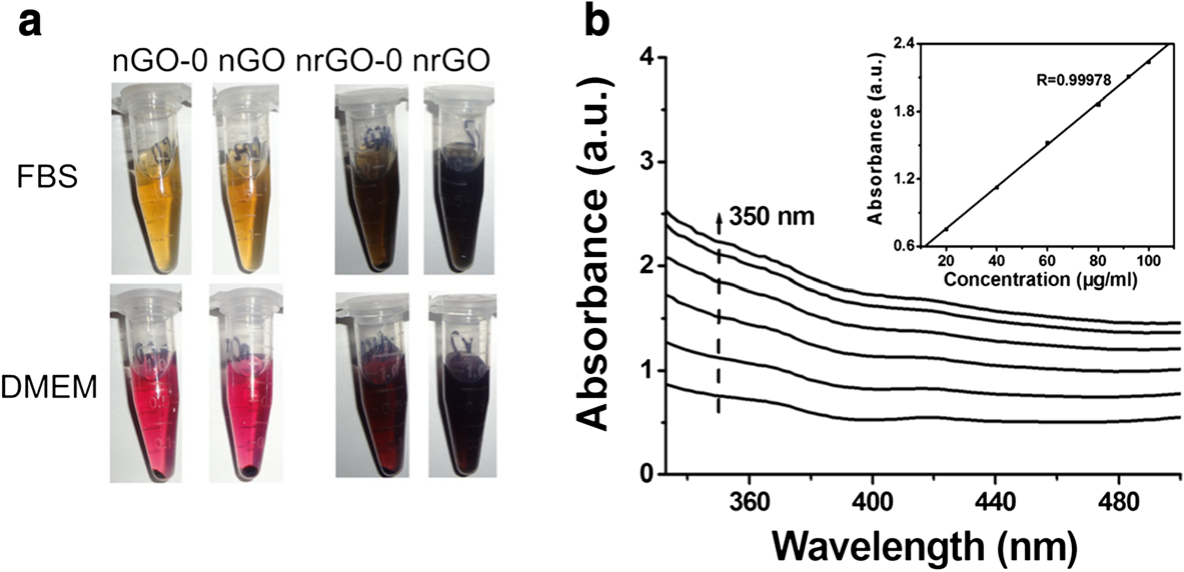
Dispersibility of nrGO. **a** Photographs of nGO and nrGO solutions in FBS and DMEM at the concentrations of 100 μg/mL after storage at 4 °C for 1 month. **b** Absorption spectra of nrGO dispersed in DMEM. *Inset*: correlation of absorbance at 350 nm against concentration

In order to confirm the dispersibility of GO materials in aqueous solution, we measured their mean diameter, polydispersity index (Pdi) and zeta potential by dynamic light scattering (DLS), and found that nGO and nrGO in water had a stable diameter (Additional file 1: Figure S1A) and Zeta potential (Additional file 1: Figure S1B) as well as Pdi (Additional file 1: Figure S1C) for 2 months. The large amount of glucose we used may act as not only a reductant but also stabilizer in consideration of the aggregation tendency of reduced GO without dispersant [4, 5]. We also evaluated the dispersion of these GO materials in fetal bovine serum and DMEM containing 10 % of fetal bovine serum, and found that all the samples except for nrGO-0 remained stable in fetal bovine serum for 1 month at 4 °C (Fig. 3a upper), and that in DMEM, only nrGO kept stable for 1 month (Fig. 3a lower), suggesting that it was the nitronium oxidation of graphite oxide and subsequent glucose reduction that endowed nrGO stability in DMEM. Absorbance spectrum was employed to further verify the good dispersibility of nrGO in DMEM (Fig. 3b). If a homogeneous solution is formed, the absorbance should be in a linear relationship with the concentration on the basis of Beer’s law [37]. The insets of Fig. 3b showed that there were good linear relationships (with *R* = 0.99978) between the absorbance at 350 nm and the concentrations of nrGO from 20 to 100 μg/mL, demonstrating the great dispersibility of nrGO in DMEM.

**Fig. 4:**
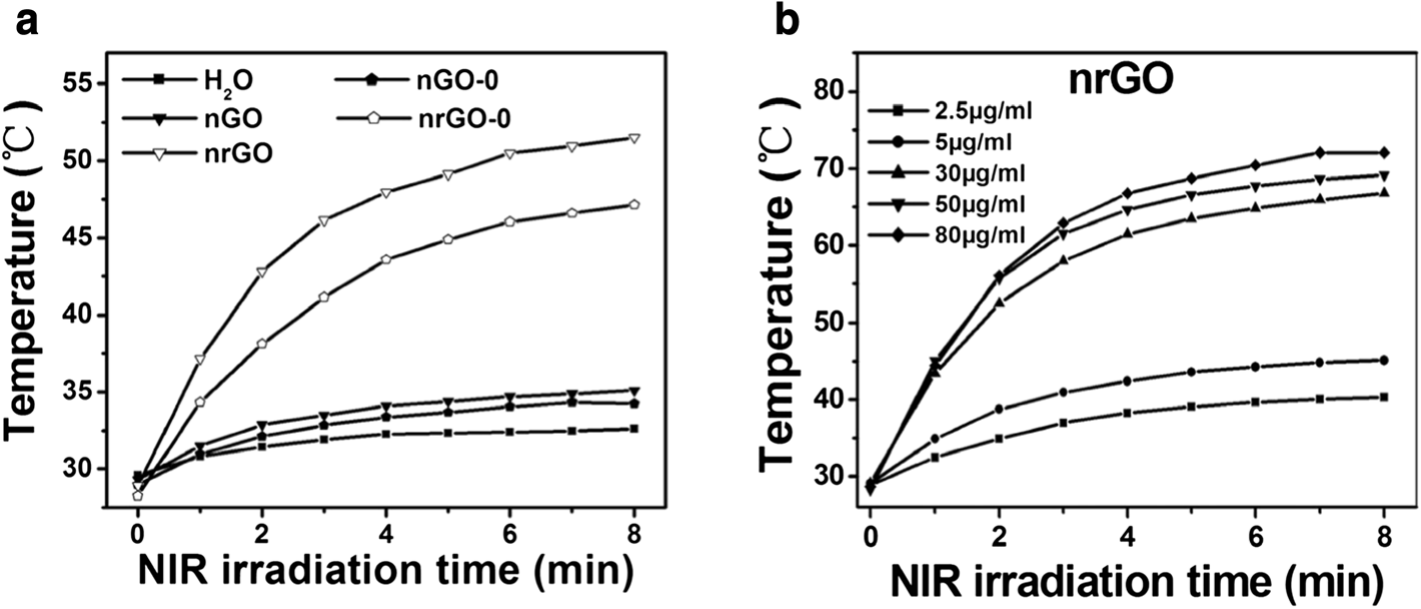
Photothermal effect of nrGO. **a** Temperature curves versus time during irradiation with 808-nm laser (3 W/cm^2^) for Eppendorf tubes containing water, nGO, and nrGO (10 μg/mL), respectively. **b** Temperature curves versus time during irradiation with 808-nm laser for Eppendorf tubes containing various concentrations of nrGO

High NIR absorbance (Fig. 2a) allows nrGO to function as an effective photothermal reagent. As shown in Fig. 4a, after 808 nm laser irradiation for 8 min, temperatures of water, 10 μg/mL of nGO-0, nGO, nrGO-0, and nrGO solutions reached ~32.6, 34.2, 35.1, 47.1, and 51.5 °C, respectively. Due to the prominent photothermal effect, nrGO was further investigated. As shown in Fig. 4b, after 808 nm laser irradiation for 8 min, different concentrations (2.5, 5, 30, 50, and 80 μg/mL) of nrGO solution showed the temperatures of 40.3, 45.1, 66.8, 69.1, and 72.1 °C, respectively.

### Characterization of Drug-Loaded nrGO

**Fig. 5:**
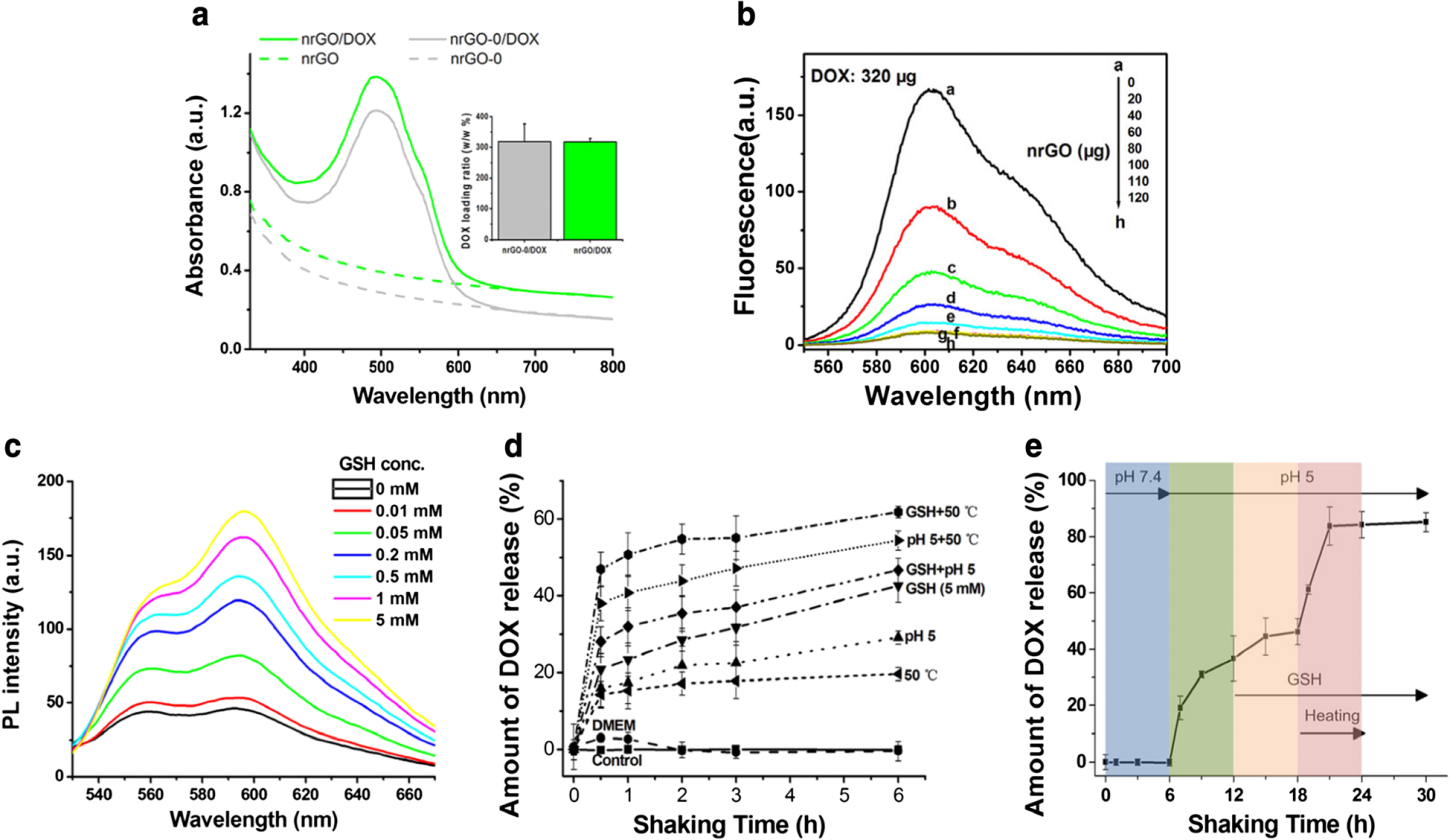
**a** Absorption spectra of nrGO (*green*) and nrGO-0 (*gray*) with (*solid*) or without (*dash*) DOX loading. *Inset*: comparison of drug loading capacity of nrGO-0 and nrGO. **b** Fluorescence spectra of DOX mixed with different weights of nrGO. **c** Fluorescence spectra of nrGO/DOX after treatment with different concentrations of GSH (0, 0.01, 0.05, 0.2, 0.5, 1, and 5 mM) for 6 h. **d** Plot of stimuli-induced DOX release from nrGO/DOX by acidic condition (pH 5), GSH (5 mM), 50 °C water bath heating, and their combinations, respectively. **e** Sequential multi-stimuli-responsive DOX release profile from nrGO/DOX complex under acidic condition (pH 5), GSH (5 mM), and 50 °C water bath heating

Doxorubicin (DOX), a broad-spectrum chemotherapeutic agent, has been known to adsorb on graphene-based materials via hydrophobic interaction and π-π stacking [5, 38, 39]. The loading ratio of DOX was 317 % (*w/w*) for nrGO and 318 % (*w/w*) for nrGO-0 by absorbance measurements after separation of the unbound DOX from nrGO/DOX solution (Fig. 5a). It was probably the abundant hydrogen bonding sites from glucose decorated on nrGO and nrGO-0 that endowed the reduced the materials’ great loading capacity [38]. DOX loading was also confirmed by monitoring the quenching of the fluorescence of DOX (320 μg) upon the addition of increasing weight (from 0 to 120 μg) of nrGO (Fig. 5b) [10, 40]. As the weight of nrGO increased, the fluorescence intensity of DOX in solution weakened gradually as a result of the non-radiative energy transfer between excited DOX and nrGO which was sensitive to the distance between them [39]. Maximum quenching was attained with 100 μg of nrGO (Fig. 5b), indicating the saturated adsorption of DOX on nrGO being at 3.2-folds of weight ratio.

### Stimuli-Triggered Drug Release

Spatially and temporally controlled drug release triggered by external stimulus is clinically significant for the effective therapy in practical drug delivery applications [10, 39, 41–44]. Here, we concentrated on stimuli-responsive release of loaded drug molecules with heating, GSH concentration, or acidic condition acting as stimulus. GSH treatment for 6 h induced a dose-dependent recovery of DOX fluorescence intensity of nrGO/DOX suspension (Fig. 5c, d), demonstrating that GSH treatment triggered effective DOX release from the nrGO/DOX complex. We also assessed the time-dependent DOX release from the nrGO/DOX complex by acidic aqueous solution (pH 5) and heating (50 °C) as well as GSH (5 mM) stimulation, and found that the nrGO/DOX complex was very stable in both aqueous solution and DMEM (supplemented with 10 % fetal calf serum), while acidic condition and heating as well as GSH stimulus triggered a rapid DOX release from the nrGO/DOX complex (Fig. 5d). Moreover, implementation of multiple stimuli stepwise to nrGO/DOX solution could induce a DOX release as high as 85 % finally (Fig. 5e).

Acceleration of drug release upon heating is likely related to change of the binding energy between nrGO and DOX [44]. Liu and co-workers reported that either 2-propanol or serum proteins induced drug release through the disruption of noncovalent π–π stacking and hydrophobic interactions between the drug and GO sheets [4]. Similarly, GSH was deemed to weaken the assembly of the aromatic interactions between nrGO and the drug [39, 44]. At low pH, DOX becomes more hydrophilic to dissociate from nrGO due to the protonation of its NH_2_ group [45]. Since nrGO owns excellent photothermal effect and the tumor is acid as well as the intracellular conditions have millimolar concentration of GSH, nrGO may thus be used as a controllable drug loading and delivery platform for cancer therapy.

### In Vitro Anticancer Effect of nrGO and nrGO/DOX

**Fig. 6:**
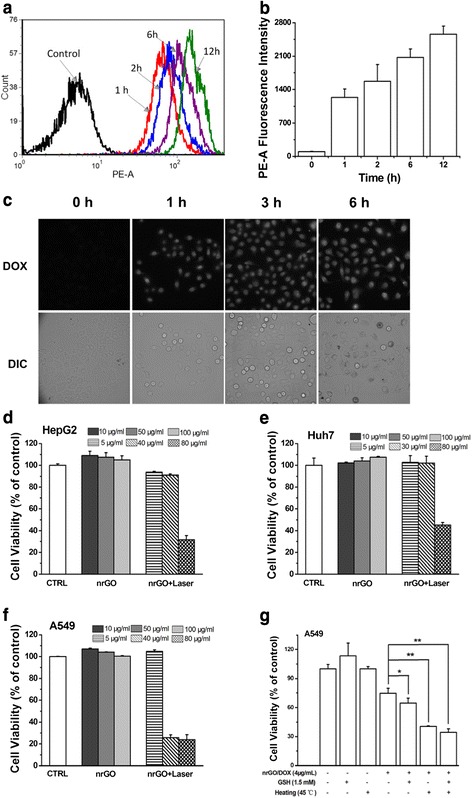
Cellular uptake and cytotoxicity of nrGO and nrGO/DOX. **a** Cellular uptake of nrGO/DOX in A549 cells by FCM assay. Cells were cultured with nrGO/DOX for 1, 2, 6, and 12 h respectively before FCM analysis. **b** Statistical results of cellular uptake of nrGO/DOX in A549 cells from three independent experiments. **c** Fluorescence images of living A549 cells cultured with nrGO/DOX. The adherent cells incubated with nrGO/DOX for 0, 1, 3, and 6 h were imaged by fluorescence microscope. **d**–**f** Dose-dependent cytotoxicity of nrGO with or without NIR irradiation on HepG2 (**d**), Huh7 (**e**), and A549 (**f**) cells. **g** Cytotoxicity of nrGO/DOX with heating or GSH treatment in A549 cells. *P < 0.05 and **P < 0.01, compared with the cells treated with nrGO/DOX alone

Considering the dispersibility of nrGO in DMEM (Fig. 3), we selected nrGO for cell experiments. Since nrGO was sterile, it was used for cells experiments directly. In order to confirm the uptake of nrGO by tumor cells, we monitored the delivery of nrGO/DOX in A549 cells just as described previously [9, 10]. After being incubated with nrGO/DOX (5 μg/ml of DOX equivalent) at different times, the cells were rinsed with PBS to remove unincorporated chemicals, and FCM analysis was applied to assess the cellular uptake of nrGO. Compared with the control group, the cells cultured with nrGO/DOX for 1 h showed strong fluorescence (Fig. 6a, b), indicating the effective uptake of nrGO by cells. Besides, fluorescent microscopy was also applied to confirm the attachment and engulfment of nrGO/DOX by cells. A549 cells incubated with nrGO/DOX for 1 h showed a strong fluorescence signal in the cell nucleus (Fig. 6c), indicating that nrGO was uptaked by cells after surface adhesion and the loaded DOX entered the nucleus successfully.

CCK8 assay was used to assess the cytotoxicity and photothermal toxicity of nrGO in HepG2, Huh7, and A549 cells. Cells cultured with nrGO alone up to 100 μg/mL for 24 h did not exhibit a decrease in cell viability whereas NIR irradiation for 5 min indeed induced approximately 68.3, 54.8, and 76.2 % decrease in cell viability for HepG2 (Fig. 6d), Huh7 (Fig. 6e), and A549 (Fig. 6f) cell lines incubated with 80 μg/mL nrGO, demonstrating the excellent PTT efficacy of nrGO to cancer cells.

Treatment with heating (45 °C) for 1 h or GSH (1.5 mM) for 2 h significantly enhanced the cytotoxicity of nrGO/DOX (Fig. 6g). It was reported that high temperature enhanced the cellular uptake of the nanomaterial possibly due to the increase in cell membrane permeability [42, 46, 47]. Kim and co-workers demonstrated that NIR irradiation effectively triggered DOX release from a PEGylated GO/DOX complex in living cells [44]. Therefore, we speculated that it was the elevated cellular uptake of nrGO/DOX and the rapid release of DOX from the nrGO/DOX complex by heating or GSH treatment that contributed to the increased cytotoxicity of nrGO/DOX.

## Conclusions

In summary, we developed a facile and rapid green approach to fabricate nanosized, sterile nrGO for photothermal therapy and drug delivery by using pure glucose as reducing agent. Of the utmost importance, nitronium oxidation of graphite oxide promotes the reduction degree and biocompatibility of glucose-reduced nrGO. In addition, the resulting nrGO has 317 % (*w/w*) of DOX loading, and the DOX release from the nrGO/DOX complex can be effectively enhanced by acid condition, GSH concentration, and heating. Taken together, the nrGO developed here may be a promising synergistic nano-platform for photothermal therapy and controllable drug delivery, which can be further applied to the nanomedicine field.

## Additional File

Additional file 1: Figure S1. Change of nGO, nGO-0, nrGO and nrGO-0 in mean diameter (Figure S1A), Zeta potential (Figure S1B) and Pdi (Figure S1C) can be found in supplementary materials. (DOC 10464 kb)

## Abbreviations

DOX: doxorubicin
FTIR: Fourier transform infrared spectroscopy
GSH: glutathione
NIR: near-infrared
nrGO: reduced nano-graphene oxide
PTT: photothermal therapy
