# Supplementary material for: Glucose-Reduced Graphene Oxide with Excellent Biocompatibility and Photothermal Efficiency as well as Drug Loading
Source: Nanoscale Res Lett. 2016 Apr 19;11:211. doi: 10.1186/s11671-016-1423-8 (PMC4837192; doi:10.1186/s11671-016-1423-8)
Supplement: Supplementary file 1 — Change of nGO, nGO-0, nrGO and nrGO-0 in mean diameter (Figure S1A), Zeta potential (Figure S1B) and Pdi (Figure S1C) can be found in supplementary materials. (DOC 10464 kb) [file 11671_2016_1423_MOESM1_ESM.doc]

**Additional file**

**
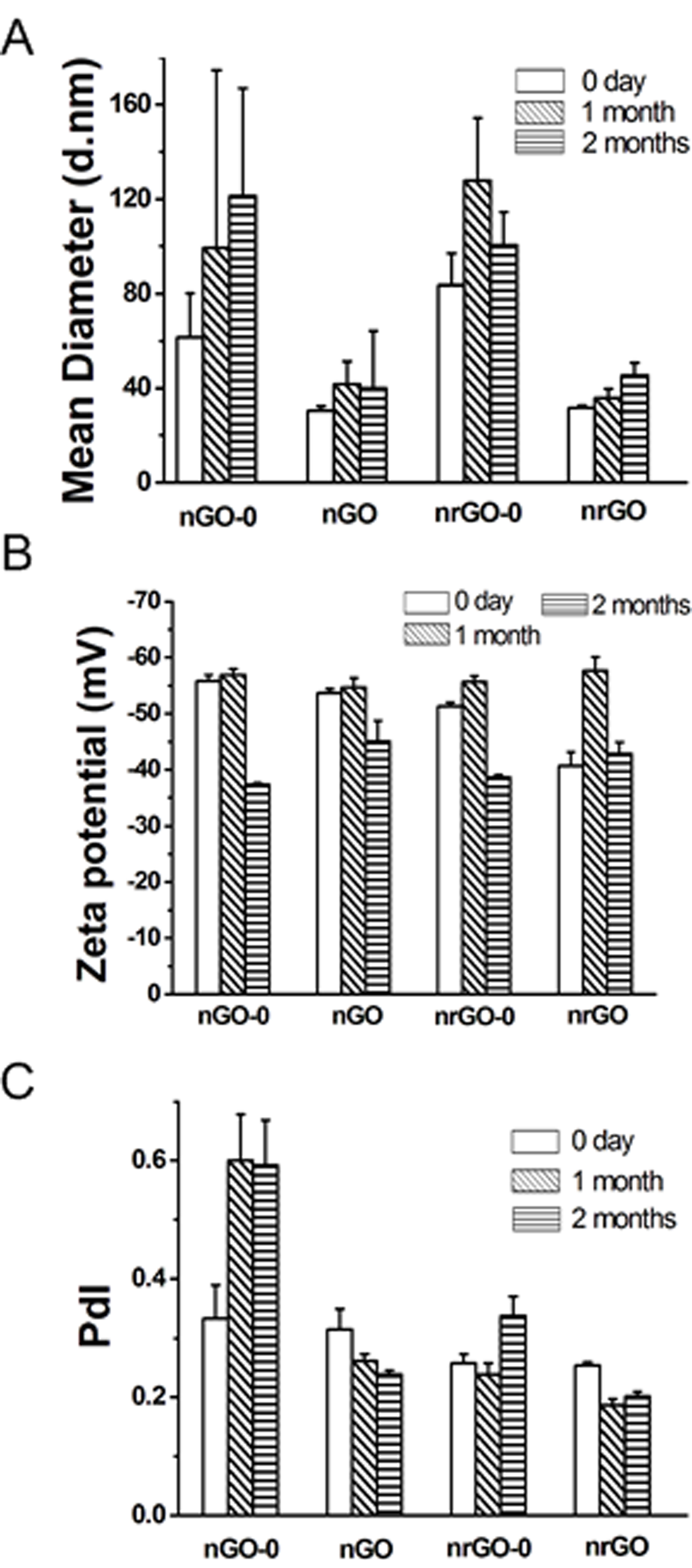
**

**Fig. S1.** Size (A), Zeta potential (B) and polydispersity index (Pdi) (C) of nrGO in aqueous solution for 2 months conducted with the Zetasizer (Malvern Instruments Ltd., UK), in which mean size =Size class (nm) * Number Distribution Date (%).
